# Diagnosis and treatment of thyroblastoma: a case report and review of literature

**DOI:** 10.3389/fonc.2025.1467631

**Published:** 2025-02-11

**Authors:** Xiting Chen, Lijuan Xiong, Hongling Liu, Haoqiang Wang, Donghai Cheng, Wei Wang, Wenyuan He, Bo Xie, Juan Zhou

**Affiliations:** ^1^ Guangzhou University of Chinese Medicine, Guangzhou, Guangdong, China; ^2^ Department of Oncology, General Hospital of Southern Theater Command, Guangzhou, Guangdong, China; ^3^ Department of Pathology, General Hospital of Southern Theater Command, Guangzhou, Guangdong, China

**Keywords:** DICER1, thyroblastoma, thyroid neoplasms, thyroid, diagnosis and treatment

## Abstract

**Background and objective:**

The diagnosis of thyroblastoma initially identified as a thyroid malignant teratoma was subsequently classified as a distinct entity by the World Health Organization (WHO) in 2022. This classification was based on the observation that the tumor presents with independent primitive multilineage elements and is frequently associated with DICER1 hotspot mutations.The objective of this study was to explore and investigate the clinicopathologic characteristics, molecular features and treatment strategies of patients with thyroblastoma, followed by a review of the previous relevant literature.

**Methods:**

The clinical manifestations, pathological characteristics, molecular features and treatment strategies of the initial case of thyroblastoma pathologically confirmed in China were analyzed.

**Results:**

The tumor was revealed to have high invasive potential, rapid disease progression, and primitive multilineage elements of pathology, including immature thyroid epithelium, spindled mesenchymal proliferations, and neuroepithelial blastema. Next-generation sequencing (NGS) confirmed the presence of germline DICER1 heterozygous pathogenic mutation at p.G1784* in patient, accompanied by the somatic hotspot mutation at p.E1813D of the RNase IIIb domain. Despite local thyroid tumor resection, the disease continued to progress rapidly. However, chemotherapy with BEP led to a reduction in the tumor. The patient’s progression-free survival (PFS) reached 15 months following the administration of BEP chemotherapy in conjunction with local radiotherapy. The patient ultimately died of cardiac arrest resulting from the progression of the cancer thrombus to the right atrium and right ventricle.

**Conclusion:**

Although thyroblastoma has been treated as a separate entity with its distinctive morphologic and molecular characteristics, its clinicopathological features, diagnosis and treatment methods and prognosis remain poorly understood, which requires more accumulated clinical case data to provide basis for the correct diagnosis and treatment in the future.

## 1 Introduction

Thyroblastoma was formally recognized as a distinct tumor entity by the World Health Organization (WHO) in 2022. Previously frequently misdiagnosed as thyroid teratoma, the recent redefinition highlights its distinctive primitive characteristics. These include thyroid epithelium, spindle cells with rhabdomyoblastic differentiation, and immature neuroepithelium. It is often observed that this tumor presents with DICER1 hotspot mutations. Clinically, it differs significantly from other thyroid tumors. It is highly aggressive, progresses rapidly, and has a poor prognosis, with approximately 50% of patients succumbing to the disease. Globally, no more than 10 cases of thyroblastoma have been reported in medical literature to date, and no standardized treatment protocol currently exists. This article presents a comprehensive examination of the clinical and pathological characteristics, molecular basis, and the diagnostic and therapeutic trajectory of a newly diagnosed thyroblastoma with DICER1 mutation. The objective of this study is to integrate findings with existing literature and contribute valuable insights for future clinical practices in the diagnosis and management of this rare malignancy.

## 2 Case report

**Figure 1:**
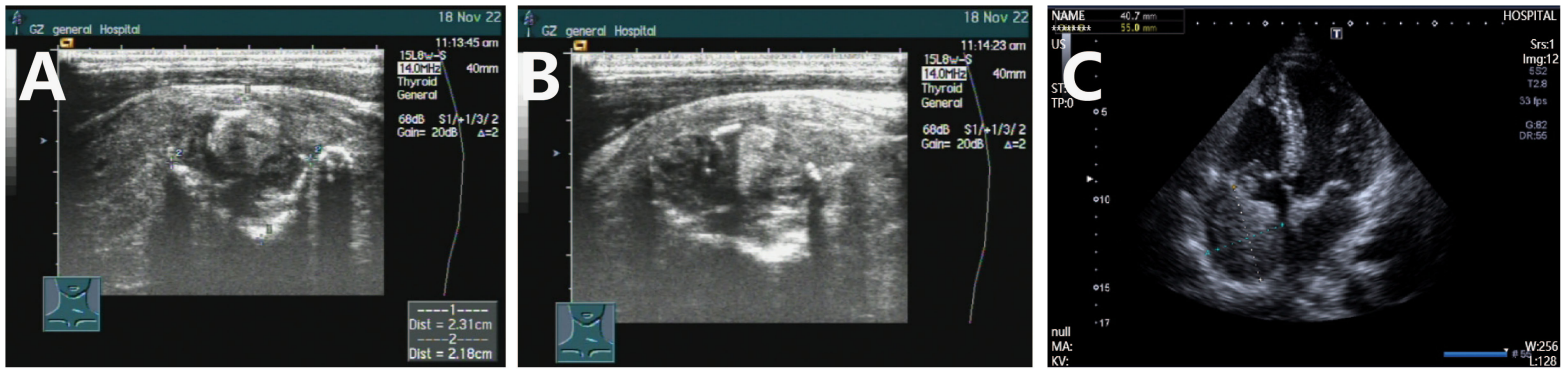
Color ultrasound examination of thyroblastoma. **(A, B)** Ultrasound results obtained on November 18 indicated an increased volume of both thyroid lobes, with heterogeneous internal echoes and multiple solid hypoechoic nodules, accompanied by calcification. **(C)** On May 20, 2024, cardiac ultrasound revealed the presence of a slightly hyperechoic mass within the right atrium, accompanied by enlargement of the right atrium and right ventricle.

**Figure 2:**
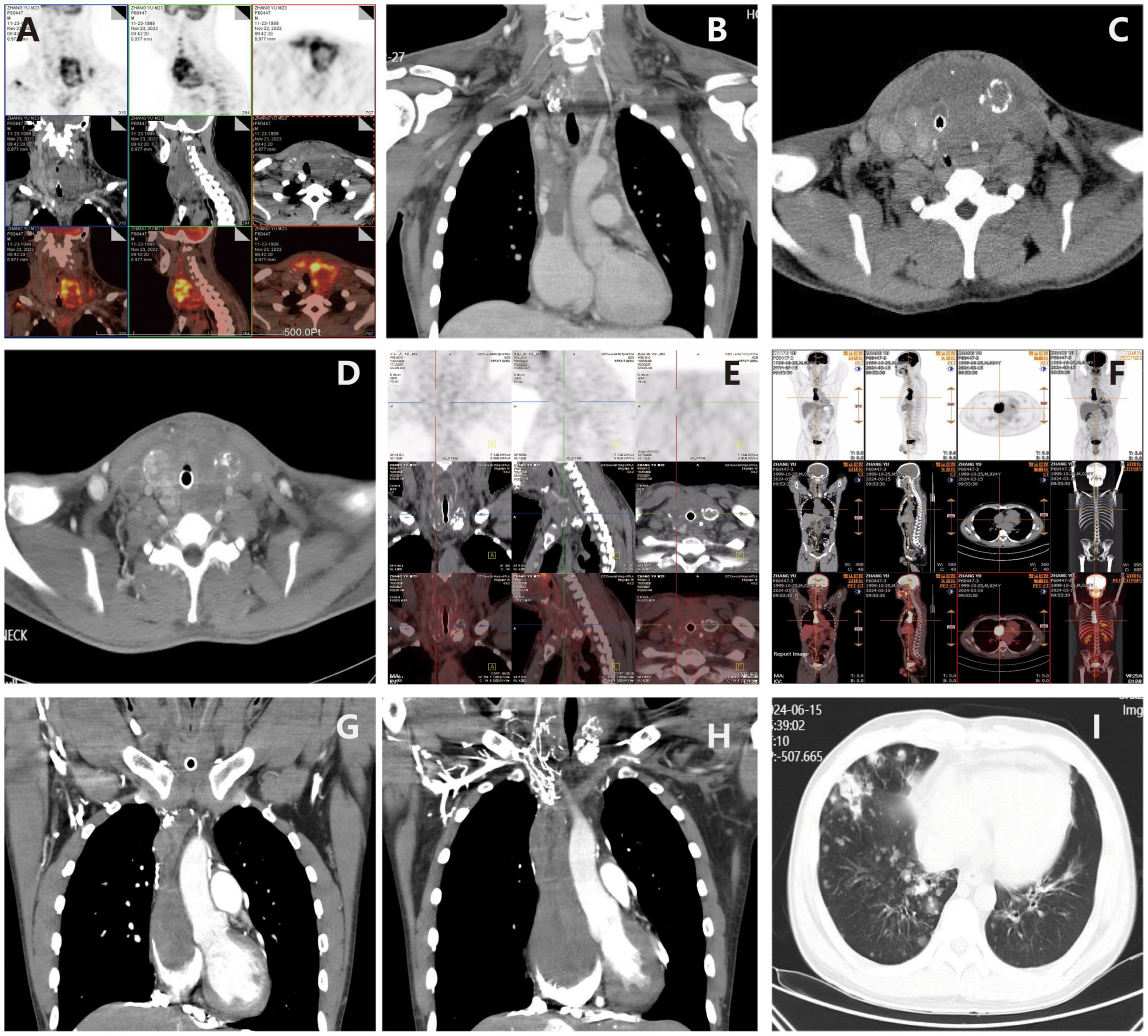
Imaging examination of thyroblastoma. **(A, B)** (November 23, 2022) PET/CT revealed irregular high-metabolic masses in the thyroid gland (more prominent in the left lobe) with calcification, compressing the trachea, causing local tracheal narrowing and rightward deviation; Multiple thromboses and cancerous thrombi were found in the superior vena cava, bilateral brachiocephalic veins, bilateral internal and external jugular veins, and the right subclavian vein. **(C)** (November 30, 2022) CT suggested rapid progression of thyroid tumor; **(D)** After two cycles of BEP regimen chemotherapy, enhanced CT (December 27, 2022) showed that the thyroid tumor had shrunk, the trachea was centered, and subcutaneous edema had decreased. **(E)** On August 25, 2023, follow-up PET/CT showed multiple low-density nodules with calcification in the thyroid, which were significantly smaller in volume than before, and metabolism had basically returned to normal, indicating that tumor activity was largely suppressed after treatment. The previously high-metabolic lesions in the superior vena cava and left brachiocephalic vein had returned to normal. **(F)** On March 15, 2024, PET/CT showed that the size of the thyroid lesion was unchanged and metabolism remained normal, suggesting suppressed tumor activity after treatment; however, new high-metabolic lesions in the superior vena cava and right atrium suggested cancer thrombi. **(G)** On March 19, 2024, CTA showed cancer thrombus formation in the lower lobe pulmonary arteries of both lungs, the right atrium, and the superior vena cava and its branches. **(H)** On June 12, 2024, CTA showed that cancerous thrombi had increased in size in the right atrium, right ventricle, superior vena cava, and left brachiocephalic vein. **(I)** On June 15, 2024, CT revealed multiple metastatic tumors in both lungs.

The patient is a 23-year-old unmarried male, visited our hospital on November 11, 2022 due to a thyroid mass that had been progressively enlarging for two months. The patient has no significant past medical history or family medical history. Upon admission, a physical examination revealed pronounced neck thickening, tracheal deviation towards the right, absence of jugular vein distension, and a palpable neck mass measuring approximately 8 cm × 6 cm. Bilateral thyroid enlargement (grade III) was observed, exhibiting a hard texture, uneven surface, absence of tenderness, and mobility with swallowing. Enlarged cervical lymph nodes were palpable, while thrill and vascular murmurs were absent. An ultrasound examination conducted at our institution on November 18, 2022, revealed the presence of multiple solid thyroid nodules, suggestive of thyroid cancer, and enlarged lymph nodes in regions II, III, IV, and VI of both neck (suggestive of metastasis) (**Figures 1A, B**). On November 23, 2022, a PET/CT scan indicated: 1. An irregular hypermetabolic mass was observed in the left lobe of the thyroid with calcification, suggestive of malignancy. This mass was found to be compressing the trachea, resulting in local tracheal stenosis and deviation to the right; 2. The presence of multiple hypermetabolic enlarged lymph nodes in the left neck (levels II-V) and mediastinum (map 1), is suggestive of metastasis; possible tumor thrombus and thrombosis in the superior vena cava and left brachiocephalic vein (**Figures 2A, B**).

**Figure 3:**
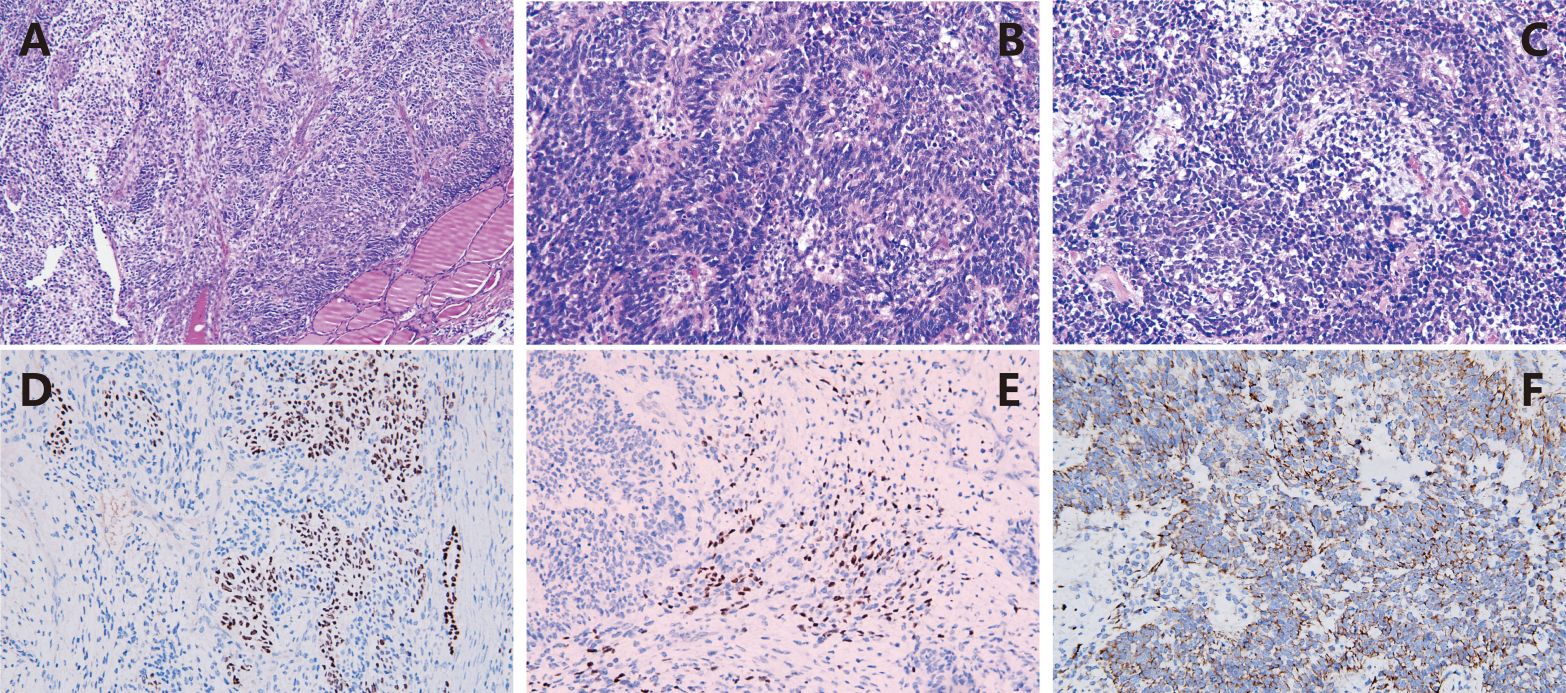
Pathological characteristics of thyroblastoma. **(A)** HE x100 The primitive tumor cells diffusely infiltrate between thyroid tissues; **(B)** HE x400 The immature cells with high nucleus-to-cytoplasm ratio are arranged in rosette-like structures; **(C)** HE x400 The tumor cells show evident apoptosis and mitotic figures, with local myogenic differentiation; **(D)** IHC x200 TTTF-1 is expressed in the primitive thyroid epithelial cells; **(E)** IHC x200 The area of rhabdomyoblastic differentiation shows immunoreactivity for Myogenin; **(F)** IHC x200 The primitive neuroepithelial components are positive for Nestin.

On November 27, 2022, due to acute respiratory distress and upper airway obstruction, the patient required emergency tracheotomy and resection of the thyroid tumor. Intraoperatively, diffuse bilateral thyroid enlargement with a hard texture and adhesion to surrounding tissues was noted, along with tracheal compression causing rightward deviation. Partial resection of the thyroid isthmus tumor was performed, and a tracheotomy tube was inserted to relieve upper airway obstruction. Postoperative pathology report (**Figure 3**) indicated that the lesion was situated within the thyroid tissue. The tumor exhibited components of primitive multipotential origin, including thyroid epithelial, spindle stromal, and primitive neuroepithelial elements. The tumor tissue within the thyroid exhibited immature cells with a high nuclear-cytoplasmic ratio, forming daisy-shaped cluster structures, large nuclei with coarse chromatin, and frequent apoptosis and mitosis. The immunohistochemistry results indicated the following findings: Thyroid epithelial components exhibited the following characteristics: SALL4(+), TTF-1 partial (+), P16(+), PAX-8 weak (+), CD99 partial (+); Immature neuroepithelial components: Syn partial (+), NSE(+), Nestin(+) and CD56(+); Stromal rhabdomyoblastic components: vimentin(+), desmin(+) and myogenin (+); Other markers: TG(-), CT(-), CK7(-), CK19(-), CK20(-), MC(-), Galectin-3(-), BRAFV600E(-), CK(-), EMA(-), S-100(-), SOX10(-), H3K27Me3(-), SMA(-), CD117 weak (+), inhibin(-), CgA(-), WT-1 cytoplasmic (+), HMB45(-), Melan-A(-), INI-1 intact, Ki67 positive rate approximately 60%(+). The diagnosis of thyroblastoma was made on the basis of the tissue morphology and immunohistochemistry results. NGS gene mutation testing revealed a heterozygous germline pathogenic mutation in the DICER1 gene p.G1784* in peripheral blood leukocyte DNA. Furthermore, a somatic mutation DICER1 gene p.E1813D was identified in plasma-free DNA, which is a hotspot mutation in the DICER1 gene RNase IIIb domain (**Table 1**).

**Table 1:** NGS results of patient tissue specimens.

Gene	Chromosomal Coordinate	Exon	Nucleotide Change	Amino Acid Change	Zygosity	Frequency	Significance
DICER1	chr14:95557628	exon25	c.5439G>T	p.E1813D	Heterozygous	13.33%	Yes
DICER1	chr14:95560239	exon24	c.5350G>T	p.G1784*	Heterozygous	47.50%	Yes
ASXL1	chr20:31019399	exon9	c.896G>A	p.G299D	Heterozygous	48.80%	No
ERCC4	chr16:14042201	exon11	c.2748G>C	p.K916N	Heterozygous	49.80%	No

Following surgery, the patient initially experienced temporary relief from respiratory distress for three days. However, subsequent CT indicated rapid progression of the thyroid tumor (**Figure 2C**). Fiberoptic bronchoscopy confirmed the growth of the tumor, which had compressed the airway, necessitating the placement of a tracheal stent. On December 6, 2022, the patient underwent emergency BEP chemotherapy (bleomycin, etoposide, cisplatin), resulting in the alleviation of respiratory distress symptoms post-treatment. A follow-up CT scan conducted on December 27, 2022, demonstrated a reduction in the size of the thyroid tumor (**Figure 2D**), prompting the continuation of the second course of BEP chemotherapy. From January 2023 to April 2023, the patient received four subsequent courses of BEP chemotherapy, achieving partial remission (PR) as the best response during this period. From April 12, 2023, to May 31, 2023, the thyroid tumor and cervical lymph node area were subjected to radiotherapy at a dose of 60Gy/30F. After radiotherapy, the tumor exhibited further reduction in size but did not achieve complete remission (CR).

**Figure 4:**
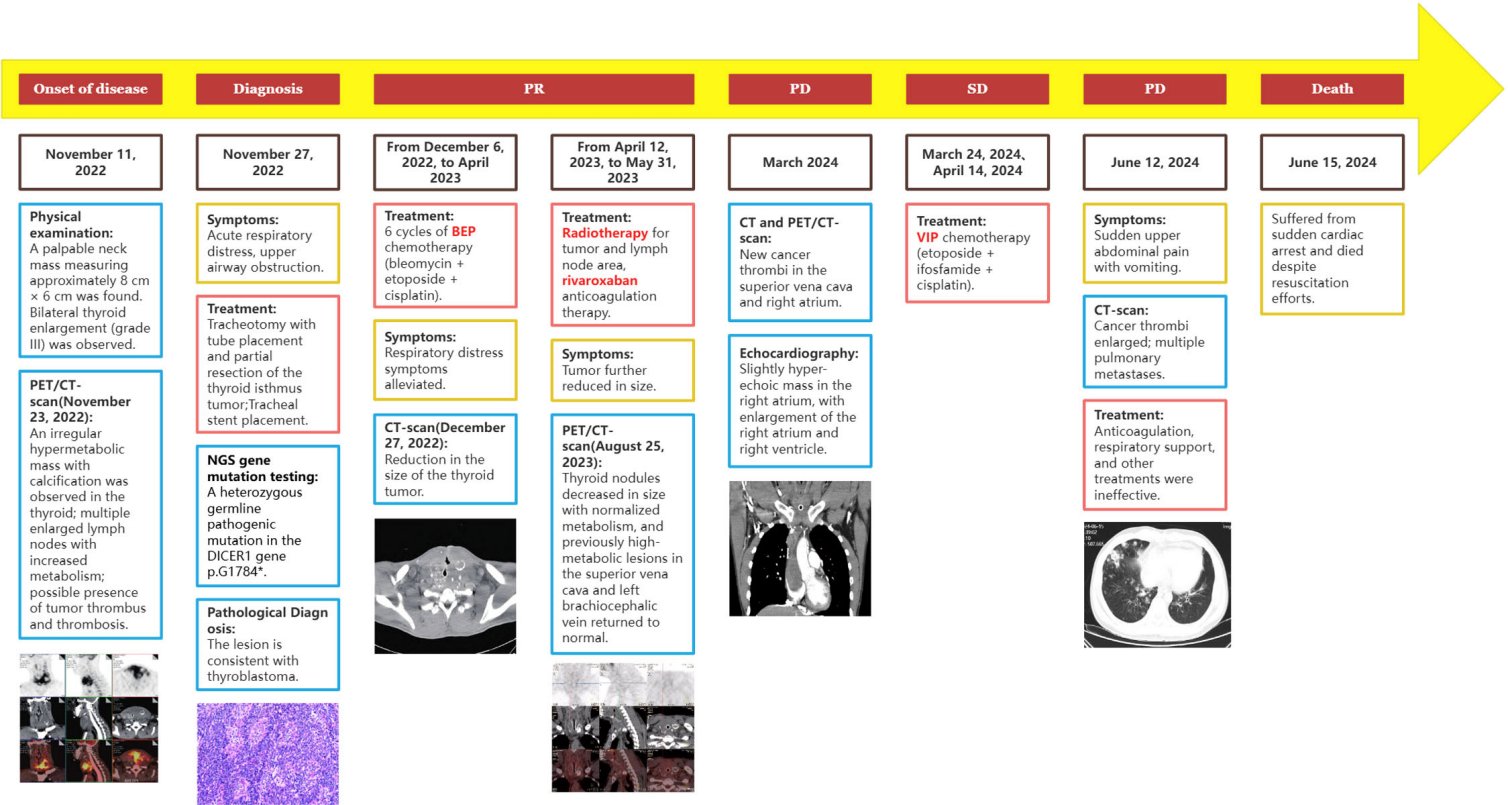
Timeline of the patient’s disease progression, diagnosis, and treatment.

After treatment, the patient was administered rivaroxaban for anticoagulation and underwent periodic follow-up examinations. The condition remained stable (**Figure 2E**). In March 2024, the patient presented to our department due to sudden syncope. The CT and PET/CT findings indicated multiple hypodense thyroid nodules with calcification, which was consistent with previous observations and indicative of essentially normal metabolism. These findings suggest inhibited tumor activity post-treatment. However, new hypermetabolic foci were detected in the superior vena cava and right atrium, suggesting the presence of a tumor thrombus. Computed tomography angiography (CTA) confirmed partial arterial involvement in the lower lobes of both lungs, right atrium, superior vena cava, bilateral brachiocephalic veins, internal and external jugular veins, and subclavian veins. Multiple collateral circulations were observed (**Figures 2F, G**). Cardiac ultrasound revealed the presence of slightly hyper-echoic masses within the right atrium, accompanied by enlargement of both the right atrium and right ventricle (**Figure 1C**). On March 24, 2024, and April 14, 2024, the patient received VIP chemotherapy (etoposide, ifosfamide, cisplatin). On May 13, 2024, the patient presented with a fever, cough, and respiratory distress, and was unable to assume a recumbent position. The follow-up CT scan indicated that the postoperative changes in the thyroid remained consistent, and the multiple tumor thrombi in the right atrium and superior vena cava were unchanged. The patient was treated for bilateral pneumonia with anti-infective agents, spasmolytics, and bronchodilators, resulting in clinical improvement and subsequent discharge. On June 12, 2024, the patient presented to the emergency department with sudden upper abdominal pain and vomiting. A CT scan revealed enlargement of tumor thrombi in the right atrium, right ventricle, superior vena cava, and left brachiocephalic vein, along with multiple pulmonary metastases (**Figures 2H, I**). After receiving anticoagulation and respiratory support, the patient’s condition did not improve. Despite resuscitation efforts, the patient suffered a sudden cardiac arrest and died on June 15, 2024. A timeline chart has been created to illustrate the progression, diagnosis, and treatment of the patient’s disease (**Figure 4**).

## 3 Discussion

According to the new classification in the 2022 WHO 5th edition of the “Classification of Endocrine and Neuroendocrine Tumors” (1), this case has been diagnosed as thyroblastoma. The literature on this particular tumor type is currently scarce, both within the domestic and international contexts. Previously classified as malignant thyroid teratoma, it is known that teratomas often occur in the reproductive system, while those occurring in the thyroid are very rare. Benign and immature teratomas mainly occur in infants, young children, and adolescents with a good prognosis, whereas malignant teratomas are more common in adults and have an aggressive course. The significant differences in clinical progression and prognosis suggest that benign/immature teratomas and malignant teratomas are completely independent solid tumors, rather than variations within a single tumor type. Thyroblastoma is primarily composed of primitive multipotential components. Thyroid follicles appear small and round, with scant cytoplasm and ovoid nuclei. Glial tissue is rare or absent, and some follicles may fuse, forming a microfollicular pattern. Spindle cell components typically exhibit a primitive undifferentiated state, with occasional short spindle and stellate shapes, often accompanied by significant mucinous degeneration and capillary proliferation. Some spindle cells exhibit rhabdomyoblastic differentiation (2–5). In contrast, benign/immature teratomas exhibit a broader spectrum of tissue components, including squamous epithelium with adnexal structures, respiratory epithelium, gastrointestinal epithelium, pancreatic parenchyma, hepatic parenchyma, pulmonary parenchyma, smooth muscle, skeletal muscle, cartilage, bone, and fibroadipose tissue. Immunohistochemistry resultsindicate the presence of immature neural elements, spindle cells exhibiting rhabdomyoblastic differentiation, and thyroid follicular-like epithelium in malignant teratomas (2, 6, 7). Typically, Pan-CK, TTF-1, and PAX8 are positive, with partial positivity for TG. Myogenic markers like Desmin and Myogenin are expressed in most spindle-shaped stromal cells, while S-100, CD99, TTF-1, and p63 may vary in expression. Primitive neuroepithelial components such as SALL4, NSE, Nestin and Syn are generally positive, with occasional positivity for Glypican-3 and TTF-1. In this case, the thyroid tissue lesion comprised multiple primitive multipotential components, including thyroid epithelial components, spindle stromal components, and primitive neuroepithelial components. Dual positivity for TTF1 and PAX8 suggests an origin from thyroid epithelium. Additionally, abundant undifferentiated spindle stromal cell components with rhabdomyoblastic differentiation were observed.Thyroblastoma needs to be differentiated from the following tumours: (1) Undifferentiated carcinoma of the thyroid (mesenchymal carcinoma): Undifferentiated carcinomas can show heterogeneous differentiation overlapping with that of thyroblastoma, including rhabdomyoblastoid cells or cartilaginous components. However, mesenchymal thyroid carcinomas occur in the elderly, usually show more pronounced cellular pleomorphism, and rarely have DICER1 mutations. Tumour cells are positive for CK, vimentin and PAX8, mutant p53 is often diffusely and strongly positive, TTF-1 is rarely positive, and neuroepithelial markers (Syn, CD56 and Nestin, etc.) are often negative (8); (2) Hypofractionated thyroid carcinoma (Insular carcinoma): Not only island-like, trabecular and microfollicular structures can be seen in poorly differentiated thyroid carcinoma, which overlap with immature fused follicles of thyroblastoma, but also some of poorly differentiated thyroid carcinomas that occur in children or adolescents have DICER1 mutations, which need to be differentiated. Hypofractionated carcinomas lack the polyblastic component of thyroblastoma and are negative for neuroepithelial labelling by Syn and Nestin (9).

The defining molecular alteration in thyroblastoma is the acquired DICER1 hotspot mutation. The DICER1 gene, located on chromosome 14q32.13, encodes an RNase III family endoribonuclease, which is responsible for processing microRNA precursors into mature microRNAs. In thyroblastoma, mutations typically occur in specific codons of the RNase IIIb domain, such as E1705, D1709, G1809, D1810, and E1813 (10). These missense mutations disrupt normal microRNA synthesis and expression, thereby leading to tumorigenesis. DICER1 syndrome, a rare autosomal dominant familial tumor predisposition syndrome, that often manifests in infants and children under 30 years of age, is associated with heterozygous germline DICER1 mutations (11). The global incidence of DICER1 syndrome remains uncertain, characterized by diverse clinical manifestations where patients may develop one or multiple related tumors. Variability is notable even within families, with different members presenting different tumor types. Common DICER1-associated tumors include pleuropulmonary blastoma, ovarian Sertoli-Leydig cell tumor, and various thyroid diseases such as papillary, follicular, poorly differentiated carcinomas, and multinodular goiter. Less common tumors encompass nasal chondromesenchymal hamartoma, ciliary body medulloepithelioma, and pineoblastoma (12). DICER1 germline mutations outside hotspot regions, and a “second hit” - somatic mutations in the DICER1 RNase IIIb domain, contribute to the development of both benign and malignant tumors. Additionally, somatic DICER1 mutations can result in comparable tumour manifestations in patients lacking germline mutations (13). In recent years, advancements in molecular detection have led to the expanding scope of DICER1-related tumors. As of 2020, primary DICER1-related central nervous system sarcoma (14) and primitive sarcoma similar to pleuropulmonary blastoma and DICER1-related renal sarcoma (15) have been recognized. Now, thyroid blastoma has been newly classified among the DICER1-related malignant tumors. Rooper et al. (16) conducted next-generation sequencing (NGS) on 8 cases of thyroid teratoma (4 malignant, 3 benign, and 1 immature), revealing a DICER1 hotspot mutations in all 4 malignant cases, while no other clinically significant gene mutations were detected in the benign and immature cases. Study revealed that all 10 published cases meeting the genetic testing criteria for thyroid blastoma exhibited DICER1 hotspot mutations, including p.E1813Q (n=1, also had p.K868Ter), p.E1705K (n=3,one also had p.Y819fs), p.D1810H (n=1), p.E1813G (n=2), p.E1813K (n=1), p.G1809R (n=1), and p.D1709N (n=1) (6, 17). Among these, 4 cases had non-hotspot mutations that inactivated other DICER1 alleles, while 2 cases had TP53 mutations, 1 case had an NF1 mutation, and another 1 case had an ATM mutation. Notably, in one case confirmed via NGS, the patient was found to harbour a pathogenic heterozygous germline DICER1 gene mutation, p.G1784*, and a somatic mutation p.E1813D. This suggests that the inherited embryonic DICER1 mutation led to a secondary somatic cell mutation and subsequent development of thyroid blastoma. Heterozygous germline mutations at p.G1784* had not been previously reported.

As the range of tumors with DICER1 mutations expands, it is worth noting that despite their appearance in various anatomical locations, these tumors exhibit similar multi-germ layer components.These components include rhabdomyoblastic differentiation, primitive neuroepithelial components, immature cartilage or bone islands, and epithelial differentiation towards the organ of origin. The histological resemblance between the multi-germ layer components of thyroid blastoma and other DICER1-related malignancies suggests that the treatment approach for thyroid blastoma could be informed by DICER1-related malignancies. The treatment of tumours associated with DICER1 syndrome primarily relies on the tumor type and surgical pathology staging, with a combination of surgery and chemotherapy being the prevalent method. The International Pleuropulmonary Blastoma Registry recommends a chemotherapy regimen consisting of ifosfamide, doxorubicin, vincristine, and dactinomycin (18). For patients with pleuropulmonary blastoma who have residual disease post-surgery, recurrence, or metastasis, radiotherapy may also be employed. The primary treatment for DICER1 syndrome-related ovarian sex cord-stromal tumors is surgical resection, often followed by adjuvant chemotherapy, typically a platinum-based combination such as BEP or PEI (cisplatin, etoposide, and ifosfamide). Although the prognosis for malignant thyroid teratomas is generally poor, Ting et al. (19) reported successful treatment in 4 patients using surgery combined with chemotherapy. Regimens included CISCA (cyclophosphamide, doxorubicin, cisplatin), BEP, POMB (vincristine, methotrexate, bleomycin, and cisplatin), and ACE (dactinomycin-D, cyclophosphamide, and etoposide). A review of the published literature reveals that the primary treatment options for thyroid blastoma include total or subtotal thyroidectomy, combined with neoadjuvant chemotherapy, and adjuvant chemotherapy with or without adjuvant radiotherapy. The most commonly employed chemotherapy regimens include paclitaxel and carboplatin (4), PEI, and EVID (etoposide, vincristine, ifosfamide, and dactinomycin) (2), and so on. Reported survival times for patients range from 10 months to 125 months, yet the specific factors influencing survival prognosis remain unclear. The chemotherapeutic regimens previously reported in the literature for thyroblastoma include paclitaxel and carboplatin, PEI (cisplatin, etoposide, and ifosfamide), EVID (etoposide, vincristine, isocyclic aminophosphate, actinomycin), etc. Thyroblastoma was often diagnosed as teratoma, which was a type of germ cell tumour, and based on the above information, we chose the BEP(bleomycin, etoposide, cisplatin) regimen, which was a commonly used chemotherapeutic regimen for germ cell tumours, and was proved to be effective in this patient. The BEP regimen has become the ‘gold standard’ of chemotherapy for malignant germ cell tumours since 1994 (20), and after more than 30 years of clinical application, the efficacy of the BEP regimen has been widely verified by clinics, and it is the recommended regimen for the guidelines of the NCCN(National Comprehensive Cancer Network) and the CSCO(Chinese Society of Clinical Oncology) guidelines, the CR rate of germ cell tumour using BEP regimen is about 80%, and the 7-year OS rate is 69%. And side effects are manageable, with lower haematological toxicity compared to VIP(cisplatin, etoposide, and ifosfamide) regimens (21). Following the diagnosis of this patient, due to the challenges of achieving complete surgical resection and the rapid tumor progression, we opted for the BEP regimen. This approach resulted in a significant reduction in the size of the thyroid tumor and improvement in respiratory function. The combination of chemotherapy with local radiotherapy resulted in a progression-free survival (PFS) of 15 months for the patient. However, significant thrombosis and cancer thrombus were present at the onset, which led to subsequent tumor progression, primarily manifesting as cancer thrombus progression. Despite effective control of the local thyroid tumors with chemotherapy and radiotherapy, the patient ultimately died of cardiac arrest caused by the cancer thrombus progression to the right atrium and right ventricle.

Thyroid blastoma is classified as a distinct solid tumor due to its distinctive morphological and molecular characteristics. **Table 2** summarizes the clinical and pathological features of all 11 published cases of confirmed thyroid blastoma, along with our current case. This new classification provides clinicians with a more comprehensive understanding of these challenging tumours, facilitating more effective diagnosis and treatment. Nevertheless, additional clinical cases are required in order to enhance our comprehension of their clinical characteristics, treatment, and prognosis.

**Table 2:** Published cases of thyroblastoma with morphologic and molecular characteristics.

Case	Sex	Age	Size(cm)	Presentation	Original Diagnosis	Treatment	Outcomes	Molecular	References
1	F	45	2.8	Neck mass	Carcinosarcoma	Total thyroidectomy, chemotherapy (taxol and carboplatin), and radiation	Lung metastasis, DOD at 11 mo	DICER1 p.E1705K	Yang et al. (4)
2	F	59	6.7	Rapidly progressing neck mass, hoarseness	Malignant teratoma	Neoadjuvant chemotherapy (primitive neuroectodermal tumor/Ewing sarcoma protocol) and total thyroidectomy	NED at > 48 mo	DICER1 p.E1813G.TP53 p.R248Q,TP53 p.Y126_splice,NF1 p.N1054H	Rabinowits et al. (3)
3	F	65	1.9	Neck mass	Malignant teratoma	Total thyroidectomy and chemotherapy (unknown plan)	NED at 125 mo	DICER1 p.E1705K, DICER1 p.Y819fs	Rooper et al. (14)
4	F	29	10	Neck mass	Malignant teratoma	Total thyroidectomy, chemotherapy (unknown plan), and radiation	Para-aortic and clavicle metastases, DOD at 53 mo	DICER1 p.E1813G, DICER1 p.V448fs	Rooper et al. (14)
5	F	42	8	Neck mass	Malignant teratoma	Total thyroidectomy, chemotherapy (unknown plan), and radiation	NED at 64 mo	DICER1 p.E1813Q, DICER1 p.K868X	Rooper et al. (14)
6	M	60	1.7	Incidental on imaging	Malignant teratoma	Total thyroidectomy, adjuvant therapy deferred because of comorbidities	Widespread skeletal metastases, DOD at 10 mo	DICER1 p.D1810H	Rooper et al. (14)
7	M	17	8.2	Rapidly progressing neck mass	Malignant teratoma	Subtotal thyroidectomy with re-excision of tumor bed, chemotherapy (cisplatin, etoposide and ifosfamide), and radiation	NED at 8 mo	DICER1 p.D1709N, TP53 p.F134L	Agaimy et al. (2)
8	F	17	6.3	Rapidly progressing neck mass	Malignant teratoma	Total thyroidectomy and chemotherapy (etoposide, vincristine, ifosfamide and actinomycin; beomycin, etoposide and cisplatin)	Mediastinal progression, DOD at 12 mo	DICER1 p.G1809R	Agaimy et al. (2)
9	F	19	5.9	Neck mass	Malignant teratoma	Surgery, radioactive iodine, external-beam radiation therapy	NED	DICER1p.Glu1705Lys, ATM variant	Tikamporn et al. (5)
10	F	45	3	Neck mass	Malignant teratoma	Surgery, chemotherapy, external-beam radiation therapy,tyrosine kinase inhibitor	DOD	DICER1 p.Glu1813Lys	Tikamporn et al. (5)
11	M	23	15	Rapidly progressing neck mass	Thyroblastoma	Excision of thyroid isthmus tumor and chemotherapy (beomycin, etoposide and cisplatin)	Sudden cardiac arrest, DOD at 19 mo	DICER1 p.G1784*, DICER1 p.E1813D	Current

DOD, dead of disease; NED, no evidence of disease.

## Data Availability

The original contributions presented in the study are included in the article/supplementary material. Further inquiries can be directed to the corresponding authors.
